# Deep-Learning-Based Indoor Human Following of Mobile Robot Using Color Feature

**DOI:** 10.3390/s20092699

**Published:** 2020-05-09

**Authors:** Redhwan Algabri, Mun-Taek Choi

**Affiliations:** School of Mechanical Engineering, Sungkyunkwan University, Suwon 16419, Korea; redhwan@g.skku.edu

**Keywords:** human following, deep learning, mobile robot, person identification, color feature

## Abstract

Human following is one of the fundamental functions in human–robot interaction for mobile robots. This paper shows a novel framework with state-machine control in which the robot tracks the target person in occlusion and illumination changes, as well as navigates with obstacle avoidance while following the target to the destination. People are detected and tracked using a deep learning algorithm, called Single Shot MultiBox Detector, and the target person is identified by extracting the color feature using the hue-saturation-value histogram. The robot follows the target safely to the destination using a simultaneous localization and mapping algorithm with the LIDAR sensor for obstacle avoidance. We performed intensive experiments on our human following approach in an indoor environment with multiple people and moderate illumination changes. Experimental results indicated that the robot followed the target well to the destination, showing the effectiveness and practicability of our proposed system in the given environment.

## 1. Introduction

In the last few years, human-following robots have become a highly active research area in various mobile robotics and computer vision applications. Computer vision technology is crucial in various practical applications such as personal guides for passengers in airports [1], elderly assistance [2], autonomous vehicles [3], surveillance systems [4], and video and sports TV [5], among others.

To follow a specific person effectively, the robot must be able to reliably track the target person within the surrounding world. However, several challenges may occur in various scenarios such as when the target person gets occluded by other people or objects due to a sudden change in his/her pose. The disappearance of the target person from the view of the robot’s camera may eventually cause the robot to stop and lose its goal. Maintaining a safe distance between the robot and the obstacles or the target in a surrounding environment without having the Freezing Robot Problem (FRP) [6] is an additional challenge for robotic follower systems. Hence, the reliable estimation of the persons’ location is quite important. Furthermore, the system must overcome all these challenges within the possibility of the available sensors (i.e., limited sensing range) mounted on the robot.

Robotic followers have been broadly studied in numerous published works and different techniques have been proposed to solve some of these challenges. One of the earliest techniques in this field used a camera to detect and track the human [7], where the authors combined a contour-based approach with a color-based approach. The human-following robot was developed using vision-based tracking for identifying an individual using the edges, color, and the texture of their clothes [8]. Bellotto and Hu [9] adopted a fusion technique using laser range data to detect the legs in order to estimate the distance to the target person and vision information to detect the faces of the people to follow the human. However, this method has a big limitation when there is occlusion by other people. Choi et al. [10] calculated the distance and angle in the screening process of children with Attention-Deficit Hyperactivity Disorder (ADHD) using simple mathematical algorithms. Li et al. [11] used a mobile robot with a red, green, blue (RGB) image segmentation process based on shape and color recognition. In [12], depth images and an H-S Histogram in a Hue, Saturation, and Value (HSV) space were obtained using a red, green, blue, depth (RGB-D) camera for service robots. Vision-based tracking for mobile robots has been proposed using template matching to follow the person [13,14]. These methods suffer from a high average computation time for video processing, thereby being sometimes insufficient for fast-tracking a human. Range-finders mounted on the mobile robot have been used for human detection and tracking due to their wide field of view (FOV) [15,16]. However, these methods have poor information about humans compared to vision-tracking methods. Previous approaches detect people using a camera or laser range-finder sensor and follow them according to their clothes’ color and positions. Although these methods try to improve the performance of human following and tracking, if the desired target is completely or partially occluded by another person or object, the mobile robot may lose track of the target person or erroneously follow another person. Therefore, the identification technique must be reliable and robust in various settings. In addition to the perceptual approach, the planning and control system should be integrated with perfect harmony to enhance the ability of the robot to deal with unexpected circumstances.

Unlike previous methods, Koide and Miura [17] integrated features of the gait, height, and appearance based on an online boosting method used as a specific human classifier. They used multiple sensors: two range finders to ensure detection of the torso and leg and a web camera to extract the clothes’ color.

Brunetti et al. [18] and Zhao et al. [19] provided a survey of the state-of-the-art human detection and tracking work by computer vision with deep learning techniques. Currently, the availability of low-cost cameras in the market allows them to be easily mounted on a robot. In modern applications, researchers use various RGB-D cameras such as Kinect, ASUS xTion and Orbbec Astra. These sensors are highly suitable for indoor environments and provide synchronized color and depth images.

In this paper, we introduce a novel framework that proposes a deep-learning-based approach for an indoor human-following robot addressing the following challenges: First, the proposed system can identify and follow the target person among other people even when there is partial occlusion of the target in the context of human-following robots. Second, the proposed system is able to perform the human-following under moderate illumination changes. Third, the proposed system attempts to recover the target in the event of the human disappearing from the robot’s FoV. This is done by selection of appropriate control for robots to maintain the robust following behavior of the person and tracking the target’s path history.

To the best of our knowledge, this is the first work that aims to continuously maintain the robot in an active state using a robust algorithm, which integrates a color feature, point cloud, and Single Shot Detector (SSD) algorithm with the Simultaneous Localization and Mapping (SLAM) algorithm for detecting multiple people and estimating all their locations using only two sensors in the context of the person identification and indoor human-following robots.

The remainder of this paper is organized as follows. First, we describe the proposed approach in Section 2. Section 3 explains the system controller of the proposed approach. In Section 4, we describe the used hardware and the experimental results that were achieved. Finally, Section 5 concludes this work and discusses some ideas for possible future improvements.

## 2. Human Following of Mobile Robot

**Figure 1:**
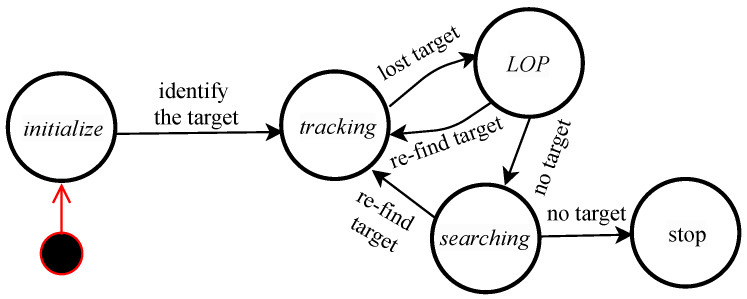
State machine diagram for the proposed human following system.

The proposed human following system is depicted in the state-machine diagram, as shown in Figure 1. This diagram describes the robotic behavior, which expresses all the different states of a robot to achieve efficient tracking and autonomous navigation in dynamic environments. The tracking state may sometimes fail to track the desired person. However, the robot must be able to recover from the failure to keep track of the target during the course. Therefore, we defined three major states, i.e., tracking, last observed position (LOP), and searching, among which the robot can switch during its operation, in addition to the initialization and stop states. If a given time is spent in a failure situation during the searching state, the robot will stop its activity by a timer or allow the users to stop it. We designed three methods in order to control the robotic behavior in each of its various states (see Section 3 for more details). In general, humans usually follow other humans in a similar manner to these methods. The first method occurs when the target person is inside the camera’s FoV, in which the robot must move directly towards the location of the person based on visual tracking. The second method takes place when the robot loses the target because the person exits the camera’s FoV, in which case the robot must navigate to the person’s LOP. The final method arises when the robot does not find the target in the LOP thus switching to the next state of searching for the target and continuing the human-following behavior around the last observed spot of the target. While these methods may not be exactly to those of people, we hypothesized that they may be close to those expected by humans for robotic behavior.

Furthermore, this work combines the SLAM algorithm [20] with a visual tracking algorithm using Robot Operating System (ROS) (http://wiki.ros.org/ROS/Tutorials) tools. All these major states are described in detail in the following sections.

### 2.1. Initialization State

In this state, the initial position of a robot [0,0,0] and its quaternion [1,0,0,0] are referenced to the base coordinate frame, where the initial vector [0,0,0] represent $\left[x_{0},y_{0},z_{0}\right]$ and the quaternion represents [orientation, roll, pitch, yaw]. In wild navigation, only 2D plane information is needed, namely the orientation ($\theta_{r}$) and coordinates ($x_{r},y_{r}$) of the mobile robot. The initialization state starts from the run moment of the code to the first frame in the tracking state, which is presented below.

### 2.2. Tracking State

Humans have several specific behavioral and physical features, such as fingerprints, iris, face and voice [21]. These features help brain processes to readily distinguish a certain person from others. Most of these features have already been studied in other fields like security. However, employing some of these features like iris and fingerprint in the robot field poses are some difficulties such as requiring direct human action from very close distances with expensive and advanced devices for identifying a certain person. However, the identification of a specific person by height, width, and clothing color is possible with affordable devices.

**Figure 2:**
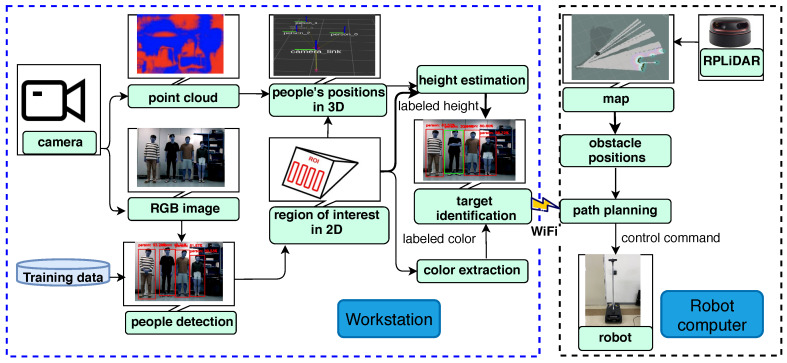
Framework of identification system.

The most important sensor of humans is arguably the eye, which detects objects by their shapes, colors, widths, and heights, among other features. Similarly, robots can use visual sensors to identify objects in the real world. The framework of the proposed method of human tracking using an SSD model and a point cloud [22] are shown in Figure 2. We implemented a novel framework that involves the outlined steps: First, the SSD was used to create bounding boxes for human detection via Convolutional Neural Networks (CNNs) in a 2D image sequence. Second, 2D coordinates are converted to 3D coordinates in world space by reducing the point cloud to the volume of the human body based on the output of the SSD detector for estimating distances. Meanwhile, the color feature is extracted from the region of interest-based also on the output of the SSD detector. Third, the distance and labeled color are obtained to identify and track the target person. Finally, this information is then sent from a workstation (node 1), which publishes the people information as a talker to a robot computer (node 2), which subscribes to this information as a listener. The robot computer is responsible for the control of the robot, path planning, and obstacle avoidance based on the publisher’s information and the surrounding environment. The ROS Master manages registration information between the ROS nodes, which is the key element in the ROS system [23]. The communication between the workstation and the robot computer is made via a WiFi connection, we used 5 GHz 802.11ac and a bandwidth of 80 MHz for consistent communication. The human tracking state is a highly complex task, especially in dynamic environments. Thus keeping high video frame rate for the human tracking is very important for the performance. Below, we briefly explain the key elements that were used in this context as follows:

#### 2.2.1. Human Detection

**Figure 3:**
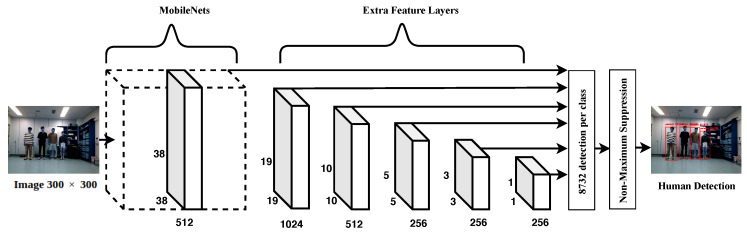
MobileNet-SSD network architecture.

**Figure 4:**
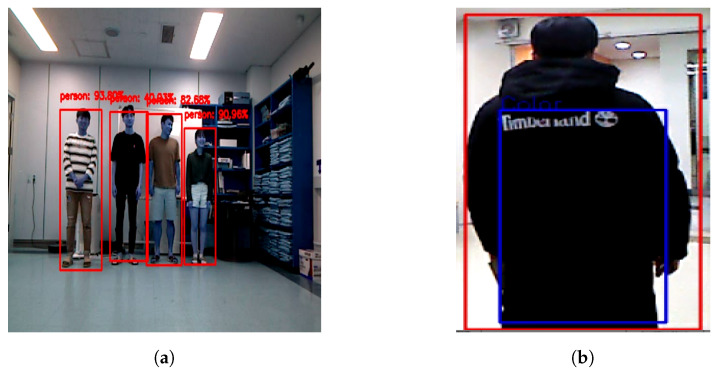
Human detection showing boundary boxes around the human’s body and region of interest. (**a**) Human detection. (**b**) Region of interest for color extraction.

To enable the robot to detect humans around it, CNNs were used by the SSD model as a detector. This technique showed excellent performance in the detection of people in real-time, thus overcoming its counterparts in detection speed. The SSD uses an end-to-end training method to improve both the speed and accuracy; it is more accurate than You Only Look Once (YOLO) [24] and faster than Faster R-CNN (regions with CNN) [25] with some data sets [26]. However, these techniques require pre-training data. Figure 3 shows the MobileNet-SSD structure. The details of MobileNets and SSD are extensive and beyond the scope of this work. Therefore, we briefly describe them here. In short, SSD achieves a balance between accuracy and speed. To detect objects using 2D RGB image sequences, in which the input size of the image is 300×300, only a single feed-forward process is required, followed by a non-maximum suppression step to produce the final detection. There are several different layers producing multi-scale feature maps and many aspect ratios with multiple scales that predict object detection. Each bounding box predicts the shape of the object with confidence, as shown in Figure 4a, where every shape is either a rectangle or a square depending on the shape of the predicted object. Therefore, every bounding box has four values in the pixel unit. They are given in the format $(u_{min}$, $u_{max}$, $v_{min}$, $v_{max})$ with MobileNets [27], (see [26,27] for more details).

#### 2.2.2. Color Feature

Color-based detection using the clothes’ color of a full-body or the upper part of a human was adopted as a key to personal identification. The color feature can be extracted easily from a sequence of images in real time. It is robust for classifying people depending on their clothes’ colors [28]. There are many methods used for object detection, depending on shape, texture or both, such as Scale Invariant Feature Transformation (SIFT) [29,30] and Histograms of Oriented Gradient (HOG) [31,32]. We used a hue-saturation histogram for its simplicity, which is one of the most popular methods for representations of color distribution. An HS-histogram is created from set pixels in a bounding box area with different sizes and positions in consecutive scenes.

In order to reduce computation time and errors, especially in the case of a colorful environment, the whole camera volume is cropped to a volume of interest, where the body is positioned. This volume in the images, which is meaningful and important, is called a region of interest (ROI). The blue box indicates the ROI, as shown in Figure 4b. The ROI is a region that concerns humans in videos or images, according to basic concepts of neuropsychological studies [33]. It is used for ease of optimization in image processing.

#### 2.2.3. Depth Image and Estimating the Distance

**Figure 5:**
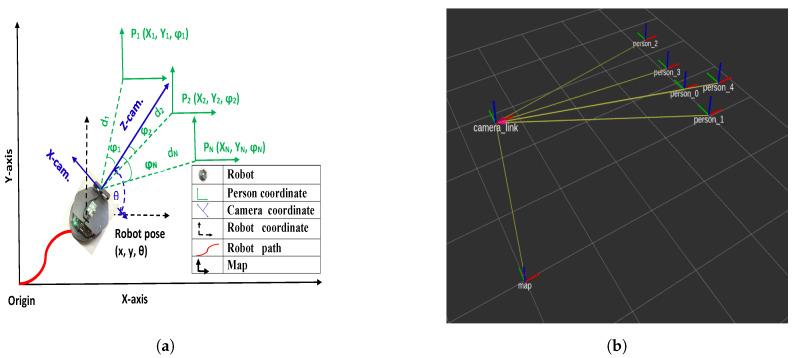
Coordinate transformation of a mobile robot. (**a**) A mobile robot mounted with a fixed camera on a 2 D plane. (**b**) A view of the standard transform library (tf) tree in the tf rviz plugin.

To avoid the FRP while switching between states presented in [34], the robot must be aware of its location and the relative positions of the people surrounding it. In human-following robot applications, estimating the accurate pose of people in 2D image data is not enough. Therefore, 3D pose estimation is indispensable. Although the CNN was originally prepared for image processing, it also became a significant component in the 3D object recognition process. The distance is not measured directly by the RGB-D images. However, if the center points of the object and camera are known in a 3D space position, the distance can be easily obtained via directly generating the coordinates of people using a point cloud. Once the SSD model detects the human’s body using a 2D RGB image sequence and creates a bounding box around the person’s body, the center of a bounding box $\left(c_{u},c_{v}\right)$ can be obtained using its vertices. The coordinate of this point can be set as the pose and the volume of interest can be extracted from the full image volume and converted from 2D-to-3D coordinates. The purpose of the 2D-to-3D conversion process is to generate a coordinate for every person based on the point cloud in meter units (see Figure 5).

Consider a group of people standing in front of the robot camera $P_{i}$, i=1,2,3,…,N, and the RGB-D camera tracking them in real-time over all frames. Let the bounding box of a person be denoted by $person_{i}$ corresponding to the first frame. This means that we simultaneously obtain *N* people, *N* centers of boxes, *N* coordinates, *N* distances, and *N* bounding boxes in a video stream. This is one powerful advantage of SSD detector and ROS for object detection. Let $person_{i}=\left\{person_{1},person_{2},person_{2},\ldots ,person_{N}\right\}$, then, the centers of the boxes are as follows: $\left(c_{u_{i}},c_{v_{i}}\right)=\left(\left(c_{u_{1}},c_{v_{1}}\right),\left(c_{u_{2}},c_{v_{2}}\right),\left(c_{u_{3}},c_{v_{3}}\right),\ldots ,\left(c_{u_{N}},c_{v_{N}}\right)\right)$, etc. The centers of boxes on the x-axis and y-axis are given by

(1) $$c_{u_{i}}=\left(u_{max_{i}}-u_{min_{i}}\right)/2$$

(2) $$c_{v_{i}}=\left(v_{max_{i}}-v_{min_{i}}\right)/2$$

where $u_{max},\;u_{min},\;v_{max}$, and $v_{min}$ are the values of the vertices of the bounding box relative to the resolution of images in each frame in terms of pixel units.

Our system adopts [35,36,37] to estimate the distance in real-world dimensions and [38] for transferring the resulting dimensions and combining them with the SLAM algorithm. In the pinhole camera model, the relationship between the camera’s image plane coordinates $\left(u_{i},v_{i}\right)$ and the 3D coordinates of people $\left(X_{i},Y_{i},Z_{i}\right)$ are relative to the camera frame, as expressed by the following equation,

(3) $$\left[\begin{array}{c} u_{i}\\ v_{i}\\ 1 \end{array}\right]=\left[\begin{array}{ccc} f_{x} & s & u_{0}\\ 0 & f_{y} & v_{0}\\ 0 & 0 & 1 \end{array}\right]\left[\begin{array}{c} X_{i}\\ Y_{i}\\ Z_{i} \end{array}\right]$$

where $f_{x}$ and $f_{y}$ are the focal lengths of the camera per axis in pixels. $u_{0}$ and $v_{0}$ are the principal point offsets of the camera per axis in pixels, (u,v) are the image coordinate axes in pixel units and *s* is axis the skew, which only appears if the *u* axis is not perpendicular to the *v* axis.

The aim of the depth information is to directly obtain the values of $\left(X_{i},Y_{i},Z_{i}\right)$ of the people relative to the frame of the camera fixed on-board the robot. Once these parameters are known, the relative distance from a person to the camera frame can be calculated in meter units. Distances $d_{i}$ from the camera to the $person_{i}$ are defined as follows:

(4) $$d_{i}=\sqrt{X_{i}^{2}+Y_{i}^{2}+Z_{i}^{2}}.$$

### 2.3. Last Observed Position State

The robot may lose the desired target due to, for example, sudden moves at high speeds around a corner. Prior information of the target person was recorded in the past state (i.e., tracking state) using the RGB-D camera, as mentioned above. When a target loss occurs, the robot navigates autonomously to the LOP of the desired target to re-find him/her and continue its following behavior. In order to achieve this task, the system uses the recorded last frame information about the target as input to the SLAM algorithm and a 2D pre-built map. Laser-based SLAM is adopted to create a depth map using a gmapping package as a ROS node called slam_gmapping (https://wiki.ros.org/gmapping), which has been proposed by Grisetti et al. [39].

If it can re-find the desired target, the robot switches to the tracking state in which the target person is within the camera’s FoV. If it fails to find the target person, the system switches to the searching state, which is presented below.

### 2.4. Searching State

The previous state assumes that a robot could re-find a person within the FoV of the camera when the mobile robot arrives at the LOP of a person. Intuitively, a person moves in several trajectories as time passes; hence, the robot may sometimes struggle to re-find and follow a person. However, the robot randomly rotates and scans the place to detect the desired target. If it re-finds the desired target, it switches to the tracking state. If the robot fails to find the desired human, such as in the event of the person moving away after a turn (i.e., corner) and disappearing, the robot will stop after a given time or allow the users to stop it. In some of these cases, it is logical to suppose that it would not be capable of detecting the desired human.

## 3. Robot Controller

To design a robust controller, we should study all the aspects of the robot navigation during all expected states. There are three major states as mentioned above: (i) the tracking state, (ii) the LOP state and (iii) the searching state. In the first two states, a proportional integral derivative (PID) controller is used [40] to reduce the error distance between the robot position and target position. In the tracking state, the target position is provided by the visual tracking. In the LOP state, the desired position is the last known position of the target before the loss of the target. The control system is a closed-loop (also known as a feedback control system). When the robot reaches the last known position of the target in the LOP, the robot moves to the searching state. While in the searching state, the robot will move randomly in an open-loop manner around the last known position to recover the target. For the safety of the robot during the random movement, we defined the maximum values of angular and linear velocities and the safe distance from the environment.

For the tracking state, the relationship between the linear velocity of the robot (V) and the error rate in the current distance, (d−D) is directly proportional, where D,d are the safe and actual distances between the robot and the target, respectively. Our system assumes *D* = 0.9 m, and *d* is the actual distance of the desired target person, which is obtained from Equation (4). The robot can navigate at speeds of up to 2 m/s and $V_{max}$= 1.08 m/s in our experiments due to safety concerns.

The relationship between the angular velocity (ω) and the error rate in the *u* coordinate direction $\left(c_{u}-u_{center}\right)$ is also directly proportional, where $u_{center}$ is the image center in the horizontal direction. The proposed system adopts $(u_{center}=\;320\;$pixel) in our experiments, and $c_{u}$ is the current center of the target in the image in pixel units, which is obtained from Equation (1). The equations of the robot velocity in the tracking state can be written as follows:

(5) $$V=k_{p}*\left(d-D\right)+K_{i}*\int_{T}\left(d-D\right)dt$$

(6) $$\omega =k_{p}^{\prime }*\left(c_{u}-u_{center}\right)+K_{i}^{\prime }*\int_{T}\left(c_{u}-u_{center}\right)dt$$

where $\left(c_{u}-u_{center}\right)$ and (d−D) are error rates for the angular and linear velocity, respectively. $K_{p},K_{i},K_{p}^{\prime },K_{i}^{\prime }$ are the PI constants and dt is the time rate between consecutive camera frames during tracking. The most difficult aspect of the PI controller is adjusting the $K_{p},K_{i}$, and $k_{d}$ values to obtain the desired behavior. In our work, we got a $K_{p}$ value of 0.415 when the distance (d) was less than 3.5 m and 0.168 when *d* was greater than 3.5 m due to safety concerns; we also got a $K_{i}$ value of 0.001. Furthermore, we got $K_{p}^{\prime }$ and $K_{i}^{\prime }$ values of 0.0023 and 0.0001, respectively.

For the LOP, proportional robot control was used to navigate to the last observed point of the target ($x_{t},y_{t}$) in the 2D plane. The pose of the robot was set as ($x_{r},y_{r},\theta_{r}$), where the angle ($\theta_{r}$) represents the robot’s heading (in radians). The angle of the target ($\theta_{t}$), from the positions of the target and the robot is determined as

(7) $$\theta_{t}=\tan^{-1}\frac{y_{t}-y_{r}}{x_{t}-x_{r}}$$

The equations of robot velocity in the LOP state can be written as follows:

(8) $$\omega =k_{p_{\omega }}*\left(\theta_{t}-\theta_{r}\right)$$

(9) $$V=k_{p_{v}}*e_{p};e_{p}=\sqrt{{\left(y_{t}-y_{r}\right)}^{2}+{\left(x_{t}-x_{r}\right)}^{2}}$$

where $k_{p_{\omega }},k_{p_{v}}$ are the proportional controller constants and $\left(\theta_{t}-\theta_{r}\right)$, $e_{p}$ are the error rates for the angular (ω) and linear (V) velocity, respectively. In all experiments, the constants were set as $k_{p_{\omega }}\;=k_{p_{v}}\;=1$.

## 4. Results and Discussion

We conducted extensive experiments for human following to demonstrate the performance of the proposed approach and tracking recovery from a failure situation when the target is lost. We divided our experiments into two categories, the first type of experiments included multi-person detection along with a target person, while the second included only one person. The visual tracking state may sometimes fail to track the target person, and the robot must be able to recover from such a failure situation. Therefore, we implemented the three major states described above, i.e., tracking, LOP, and searching. The units of coordinates, velocities, and time in all the experiments were meters, meters per second, and seconds, respectively. This section introduces a detailed explanation of the robotic behavior while testing.

### 4.1. Infrastructure Setting

**Figure 6:**
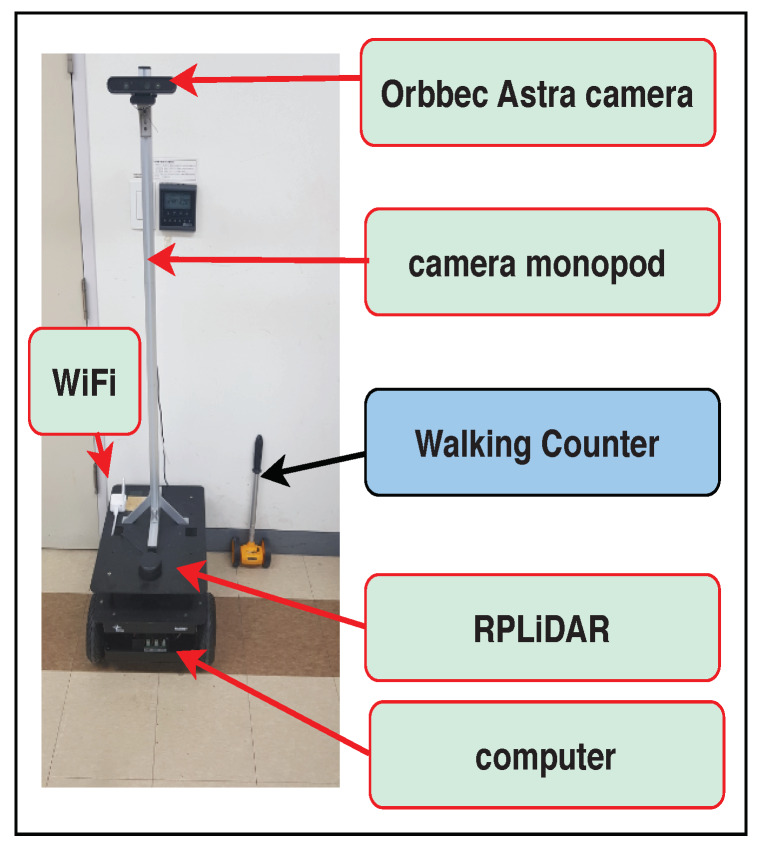
Mobile robot equipped with an RGB-D camera and range finding sensor.

The experiments reported in this paper were carried out using a robot, called Rabbot (http://edu.gaitech.hk/ria_e100/index.html), to evaluate the performance of the proposed approach, shown in Figure 6. The robot was fitted with an SLAMTEC RPLIDAR A2M8, Orbbec Astra and a computer (2.8 GHz with 4 GHz turbo frequency, i5 processor, hexa core, 8 GB RAM, and 120 GB SSD) called the robot computer, under ROS Kinetic+Ubuntu 16.04 64-bit. An Orbbec Astra camera was an RGB-D sensor that provides synchronized color and depth images. Rabbot was equipped by the camera with a resolution of 640×480 and a height modified to 1.47 m from the floor for better visual tracking of the environment. As mentioned above in Section 2.3, to create a map and obtain precise information about surrounding object positions, an RPLIDAR A2M8 was used. The code was run on two computers, one of them was the workstation for the deep learning module (Intel Core i7-6700 CPU @ 3.40 GHz) and the second one was an on-board computer in the robot. The communication between the workstation and the robot computer was made via a 5 GHz WiFi connection. Rabbot has a weight of approximately 20 kg and can carry a payload of approximately 50 kg.

### 4.2. Human Following Experiments

**Figure 7:**
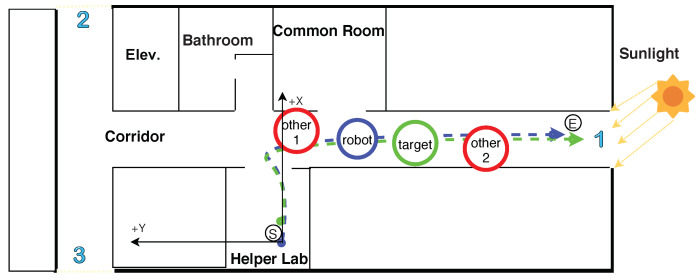
Realistic scenario of paths of the robot and the target in human following.

The testing environment is depicted in Figure 7. The target person walks along a realistic scenario path and the robot follows him/her. The path begins from the laboratory (small black circle with the letter ‘S’, which is the robot departure point) to the end of the corridor (the small black circle with the letter ‘E’, which is the target person destination point). We chose this path for testing because it is the longest and most optimal for the WiFi signal range. Its length is approximately 27 m, measured using wheel odometry, then validated with the walking counter (WC). The blue and green dashed lines represent the paths of the robot and the target, respectively. The target person walks in front of the robot and the Orbbec Astra camera tracks the target person using RGB and depth data. The red circles indicate other people, the blue numbers indicate glass walls, doors, and windows that allow sunlight to pass into the testing environment.

In the first category, the experiments included multi-person detection along with the target person in the testing environment. The performance of the proposed system was analyzed in the 18 experiments, as shown in Table 1. The system recorded data while the target walked at a normal speed. The total number of frames, the traveled distance of the robot and the time for these experiments were (18,563 frames, 450.80 m and 797.12 s), and the averages of frames, distance, and time for every experiment were (1031.3 frames, 25.04 m and 44.28 s), respectively.

The robot failed to recover the target person in two experiments for two main reasons; the first was due to the disappearance of the target person around a corner or other objects and the continuous walk toward the destination point; the second was because the robot tracked another person due to severe illumination changes, which is one limitation of the used cameras. This failure occurred after the robot traveled 18.58, 10.94 m in two failed experiments for tracking, LOP, and searching, as defined in Figure 1. However, the target person was correctly followed to the destination point during 16 of the experiments. The symbols, O,X shown in the second row from Table 1 indicate the experiment status. *O* indicates that the robot arrived at the predefined destination point and *X* indicates otherwise. Our judgment was based on success or failure, i.e., if the robot arrived at the destination point or not, regardless of the percentage of traveled distance in the failed experiment. The system was manually stopped by the observer.

In the successful experiments, the average travel distance of the robot was 26.33 m, which is comparable to the average travel distance of the target, 26.47 m. The traveled distance of the target was measured eight times using a WC. In addition, it was estimated for all experiments via the robot’s camera using a tf tree, as shown in Figure 5b. The average computation time for the video in real-time was 23.34 frame/s, i.e., 42.84 ms, which corresponds to the frame rate of the Orbbec Astra camera and is suitable for a smooth robust human-following robot. The average and maximum robot velocities were (0.57 and 1.08 m/s), respectively.

**Figure 8:**
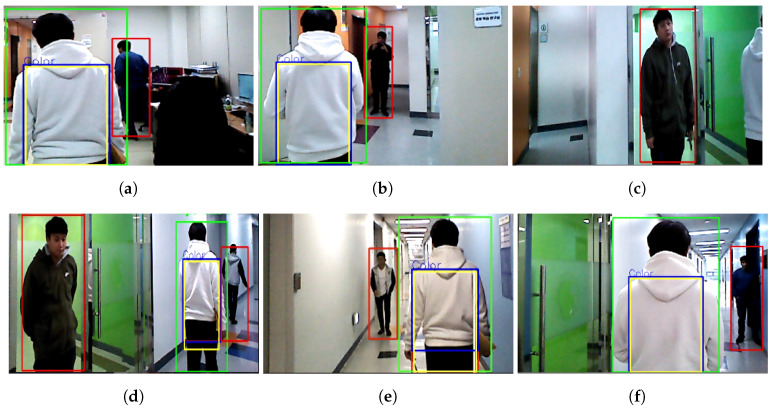
Snapshots of the robot’s field of view while performing the human following experiments: (**a**) It begins to follow the target wearing the white hooded t-shirt. (**b**) The target is walking out of the laboratory, and another person is standing in the corridor outside the door. (**c**) The robot loses the target momentarily at the laboratory’s door. (**d**) The robot re-tracks the target immediately. (**e**) The robot follows well while another person is walking in the opposite direction. (**f**) The robot follows well despite illumination changes while the other person is standing in the middle of the corridor.

Figure 8 shows snapshots of the experiments. The green bounding box around a person in the images indicates the target person and the red bounding boxes indicate the other people. The yellow rectangle on the target person indicates the ROI and the blue rectangle indicates the detection of the clothes’ colors. The test began at the laboratory and finished the end of the corridor in an indoor colorful environment. The mobile robot followed the target person based on his/her clothes’ colors.

At the beginning of the test, the mobile robot extracted the color feature of the target person and estimated his position, then started following him. A second person moved from the laboratory (Figure 8a) to the corridor (Figure 8b). During the testing, the target person disappeared behind the wall that separates the laboratory and corridor. As a result, the robot lost the target, and it simultaneously detected another person as a non-target (Figure 8c). However, the robot re-tracked the target based on the LOP and it correctly re-identified him based on the color feature. Two other people appeared in the scene, one of them stood in front of the common room while another was walking in front of the target (Figure 8d). The robot kept following the target while one person was walking forward and backward freely along the left side of the target in the corridor (Figure 8d,e), and another was standing on the right side (Figure 8f). Although there was an illumination change due to the sunlight in the testing environment, especially at the end of the corridor (see the difference in Figure 8d,e), the robot successfully followed the target person.

**Figure 9:**
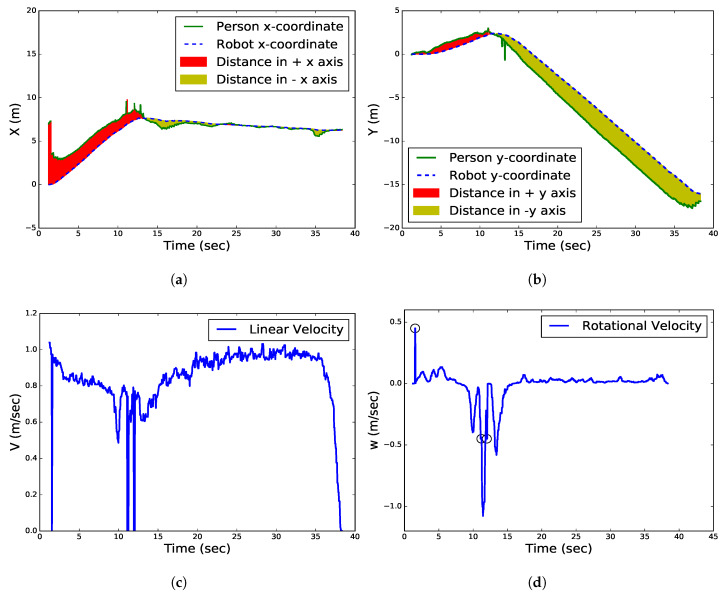
Robot and person movement trajectories in a realistic scenario for tracking state. (**a**) Relative position between the target and the robot in the x-coordinate. (**b**) Relative position between the target and the robot in the y-coordinate. (**c**) Linear velocity. (**d**) Rotational velocity.

Figure 9 shows the robot and the target person movement trajectories in a realistic scenario during the tracking state. Figure 9a,b show the positions of the robot and person along the *x* coordinate and *y* coordinate, respectively. The green curve indicates the target person trajectory while the blue dotted curve indicates the robot trajectory. The red, yellow areas between the curves of the robot and the person along the x,y coordinates indicate the distance difference (d−D) between them with +,− directions relative to a departure point (0,0) of the robot, respectively. The high frequency in the position component while rotating both the robot and person from 11 to 13.5 s at a corner between the laboratory and corridor is due to sensor noise. If we compare their trajectories with the testing environment, the target person walked in the laboratory in the +x direction, with a slight slope toward the +y direction. Then, they turned to the right at the door and kept walking in the corridor in the −y direction (see Figure 7.)

Figure 9c,d show the linear and rotational velocities, respectively. The velocity components of the robot are smooth that are well within the limits of the robot. In this experiment, the maximum and minimum linear velocities of the robot were 1.039 and 0.0 m/s at the first and last frame for the successful frames, respectively, and the average linear velocity was 0.85 m/s, while the rotational velocity was high during a rotation of the robot in the −y direction from 9.5 to 15 s at the corner between the laboratory and the corridor. When the target person walked in an approximately straight line, the rotational velocity was approximately equal to 0.0 m/s because the position of the person center was kept in the heading direction. Due to camera noise, the robot lost the target. The loss of 2, 4, and 4 frames occurred at 1.5, 11.2, and 12 s, respectively. As a result, the robot started rotating toward the LOP with a rotational velocity of ±0.45 m/s and the linear velocity dropped to zero. The black circles indicate the lost frames, as shown in Figure 9d. This means that the robot is in a persistent state of activity even when the target is lost for a millisecond. When the person arrived at the destination point, he stopped, therefore, the distance between the robot and the person began decreasing to 0.9 m. Consequently, the velocity of the robot started decreasing until it stopped (see Figure 9b,c).

### 4.3. Target Recovery Experiments

**Figure 10:**
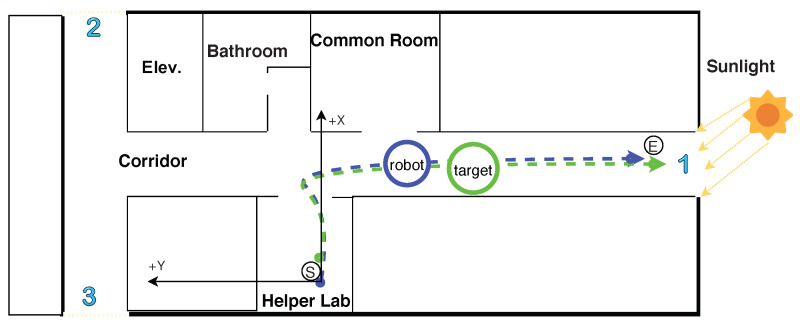
Realistic scenario of paths of the robot and the target for recovery tests.

To show the capability of target recovery, we performed different experiments. The testing environment without other people is depicted in Figure 10. It is identical to the testing environment shown in Figure 7. The path starts from the laboratory (small black circle with the letter ‘S’, which is the robot departure point) to the end of the corridor (the small black circle with the letter ‘E’, which is the target person destination point). The blue and green dashed lines represent the paths of the robot and the target, respectively.

Table 2 shows the result of the 11 experiments, which constitute the second type of experiments. These experiments include only one person in the testing environment. The total number of frames, the traveled distance of the robot, and the time for all experiments were approximately 15,562 frames, 287.9 m and 673.6 s, and the averages of frames, distance, and time for every experiment were 1414.73 frames, 26.18 m and 61.24 s, respectively. The robot failed to recover the target person in one experiment; after the robot moved to the LOP, it looked for the person in the opposite direction, as the person moved to the right and the robot moved to the left in the corridor. However, the robot recovered human tracking and continued to follow the person to the destination point in 10 experiments. The symbols, O,X shown in the second row of Table 2 indicate the experiment status. *O* indicates that the robot arrived at the predefined destination point while *X* indicates otherwise. Although these experiments were conducted with one person, the rate of successfully tracked frames decreased from 91.98 to 0.58% due to the intentional disappearance; this indicate that the robot spent a long time in the LOP and searching states. Meanwhile, the overall success rate increased from 88.9 to 90.91% due to the behavior of waiting near the LOP after the target was lost.

**Figure 11:**
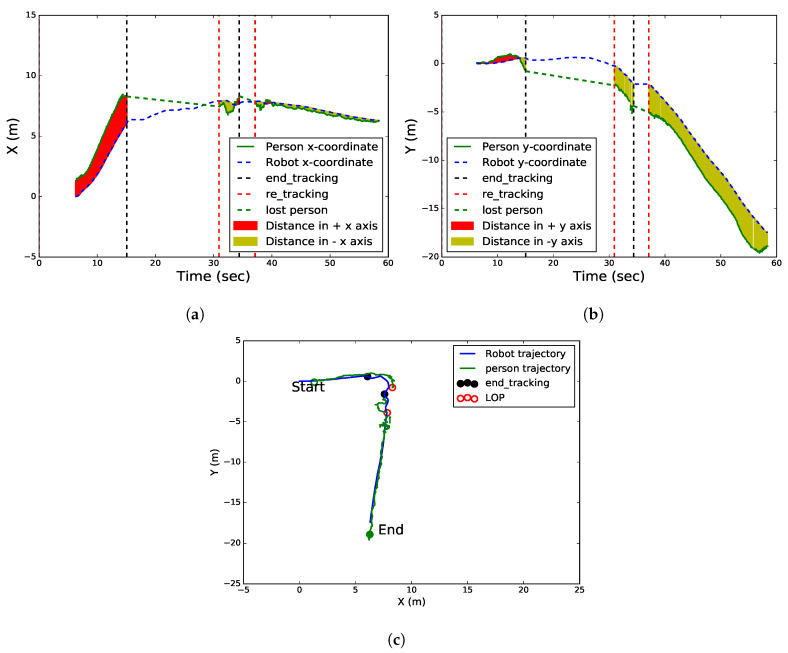
Robot and person movement trajectories in a realistic scenario for two states. (**a**) Robot and person trajectories along the *x* coordinate. (**b**) Robot and person trajectories along the *y* coordinate. (**c**) Robot and person trajectories in the world coordinate (2D) for two states.

The graphs in Figure 11 show the robot and person movement trajectories in a realistic scenario under the tracking and LOP states. Figure 11a,b show the positions of the robot and the person along the x−y coordinates. The green curve indicates the target person trajectory while the blue dotted curve indicates the robot trajectory. Black and red vertical dotted lines indicate the ends of the tracking and re-tracking, respectively. The green slanted dotted line indicates the disappearance of the target person from the robot’s FoV, as shown in Figure 11a,b. Similar to Figure 9a,b, the difference between these two figures is the disappearance of the target twice intentionally from the robot’s FoV, and the person waiting near the LOP behind the wall. However, the robot was able to re-track the target after arriving at the LOP in the first disappearance (from 15.1 to 30.7 s) while it was able to re-track the target in a short time during the second disappearance (from 34.42 to 37.1 s). When the person arrived at the destination point, he stopped, thereby, the distance between the robot and person began decreasing to 0.9 m (see Figure 11b).

The robot and person trajectories were created in the x−y coordinates by a path planner, as visualized in Figure 11c. The trajectories of both the robot and the person are almost identical. The filled black and empty red circles indicate the positions of the robot and person at the last tracked frame, respectively. The movement of the robot between them was carried out under the LOP state.

**Figure 12:**
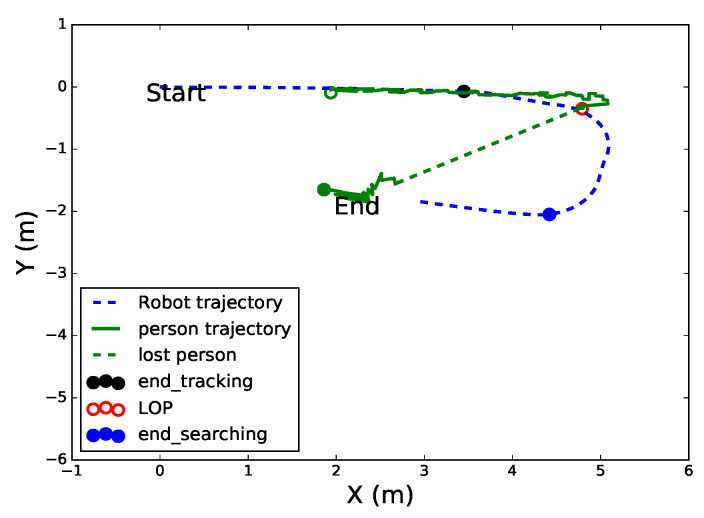
Robot and person trajectories in the world coordinate (2D) for three states.

Figure 12 shows the trajectories of the robot and person in the 2D plan for three states. The green slanted dotted line indicates the disappearance of the target person from the robot’s FoV. Similar to Figure 11c, the difference is that this experiment was conducted in the laboratory only and the robot could not find the target at the LOP; therefore, it switched to the searching state. The robot departed from the origin point (0,0) toward the target person based on visual tracking, then lost the target at (3.7, −0.084 m) where the LOP was at (4.8, −0.34 m). The robot moved to the LOP and did not find the target there due to a change in his position; it switched to the searching state to find the target, and found him where the robot was, at (4.419, −2.06 m). The filled black and empty red circles indicate the positions of the robot and person at the last tracked frame, respectively. The trajectory of the robot between them was carried out under the LOP state. The filled blue circle indicates the end of the position of the robot in the searching state and the start of the re-tracking of the target. The movement of the robot between the empty red and the filled blue circle was carried out under the searching state.

### 4.4. Comparison with Previous Approaches

As mentioned in Section 1, there have been significant researches on the subject of the human following of mobile robots. It is generally difficult to directly compare the performances of different robotic systems due to several factors such as different functional scopes, non-uniformity of sensors/hardware used, unavailability of a common dataset and non-identical operating conditions. However, in order to clarify our technical contributions, we try our best to compare our system with previous significant approaches, such as by Koide and Miura [17] and Lee et al. [41] at the function or module levels rather than the full system level.

Developed when deep learning was not common, Koide and Miura showed the full-scale human following that worked in both indoor and outdoor situations. They utilized various features such as appearance, color, height and gait, jointing features with online boosting to have a robust human following in severe illumination environments. On the other hand, Lee et al. used deep learning for indoor human following like us, adapting a sophisticated Bayesian-based target trajectory prediction. They did not show the experiments with occlusions by other people and illumination changes exclusively as much as we do.

The successful tracking rate, defined as the ratio of the number of successful frames (where the target was correctly tracked) to the total number of frames [13] is critical for successful human following. As shown in Table 1, the successful tracking rate of our indoor human following was about 92%, while Koide’s approach showed 81.0% for their outdoor following. Unfortunately, Koide and Miura did not provide the tracking rate of the indoor following in their paper. Despite the mission environments are different, our approach showed better performance for the tracking of the target during the following. Although Lee et al. had similar indoor following like us, we cannot compare because they did not provide their tracking rate.

The computation time for the overall human following that we proposed took approximately 0.042 s, as shown in Table 1. According to our findings in the paper, Koide’s method took 0.5 s, which was much longer than ours probably because they adapted multiple feature joints for both indoor and outdoor use and did not use the latest technologies like deep learning. According to our findings in the paper, Lee’s method took 0.3 s, which was also much longer than ours probably because they used Bayesian-based target trajectory prediction (a sophisticated function that we do not have yet.)

The mission success rate, showing how successfully the robot followed the target to the destination, was 88.9% in our case, as shown in Table 1. Other papers did not provide any information about the rate. The high mission success rate is important for robot developments because it shows how well the robot system is integrated for repeatability. Although we need to work harder to improve our human following, our system is fast, robust and repeatable in the event of occlusions and illumination changes.

## 5. Conclusions

This paper presents a novel framework that integrates the deep learning technique and state-machine control to develop a reliable and repeatable human-following robot. People are detected and tracked using an SSD detector, robust to occlusion. The target person is identified by extracting the color feature using an HS-histogram from a video sequence, robust to illumination changes. The robot follows the target safely to the destination using SLAM with the LIDAR sensor for obstacle avoidance. The contributions made in this work are given as follows. First, we designed an efficient and repeatable robotic state-machine control so that the robot stays in an active state of the human following without freezing. Second, we adopted a robust vision algorithm from occlusions and illumination changes integrating a color feature with the deep learning algorithm. Third, we developed a robust human following and verified it in realistic indoor situations.

Despite the high success rate in the performance of the proposed approach, it has some limitations. For instance, person identification is fully dependent on the color feature, which becomes meaningless when there are people wearing clothes that have the same or similar color. In addition, our proposed system is weak in the case of severe illumination changes. The proposed method has numerous applications for mobile robots, such as carrying luggage or belongings for the elderly or disabled or delivering goods in grocery stores or airports. For possible future improvements, we are currently working on predicting the target’s trajectories with sophisticated algorithms such as the Markov and Bayesian model instead of random search. In addition, we plan to use more human features in addition to the color, such as texture and shape, to make it robust to occlusions and illumination changes in various situations.

## Figures and Tables

**Table 1:** Experimental results of human following.

Parameters	Exp. 1	Exp. 2	Exp. 3	Exp. 4	Exp. 5	Exp. 6	Exp. 7
Mission success rate (%)	O	O	O	O	O	O	x
Target’s travel distance (m)	28.06	27.93	28.08	28.42	27.97	27.74	0.00
Robot’s travel distance (m)	25.77	26.95	26.86	27.65	26.88	26.46	18.58
Robot’s travel time (s)	44.28	42.16	43.44	45.68	42.18	42.39	68.96
Robot’s average velocity (m/s)	0.58	0.64	0.62	0.61	0.64	0.62	0.27
# of frames	1042	1024	1022	1120	932	992	1507
Successfully tracked (frames)	1030	997	1010	1108	928	982	679
Lost track of the target (frames)	12	27	12	12	4	10	828
Successfully tracked (s)	43.77	41.04	42.93	45.19	42.00	41.96	31.07
Lost track of the target (s)	0.51	1.11	0.51	0.49	0.18	0.43	37.89
Lost track of the target rate (%)	1.2	2.6	1.2	1.1	0.4	1.0	54.9
Successful tracking rate (%)	98.8	97.4	98.8	98.9	99.6	99.0	45.1
Computation time (fps)	23.53	24.29	23.53	24.52	22.10	23.40	21.85
**Parameters**	**Exp. 8**	**Exp. 9**	**Exp. 10**	**Exp. 11**	**Exp. 12**	**Exp. 13**	**Exp. 14**
Mission success rate (%)	x	O	O	O	O	O	O
Target’s travel distance (m)	0.00	27.93	27.37	27.40	28.79	27.85	28.05
Robot’s travel distance (m)	10.94	27.08	26.43	25.89	27.21	26.45	25.82
Robot’s travel time (s)	39.93	44.53	44.27	41.22	42.82	44.58	42.90
Robot’s average velocity (m/s)	0.27	0.61	0.60	0.63	0.64	0.59	0.60
# of frames	961	1042	987	999	1026	1017	991
Successfully tracked (frames)	479	969	983	994	994	941	973
Lost track of the target (frames)	482	73	4	5	32	76	18
Successfully tracked (s)	19.90	41.41	44.09	41.01	41.48	41.25	42.12
Lost track of the target (s)	20.03	3.12	0.18	0.21	1.34	3.33	0.78
Lost track of the target	50.2	7.0	0.4	0.5	3.1	7.5	1.8
Successful tracking rate (%)	49.8	93.0	99.6	99.5	96.9	92.5	98.2
Computation time (fps)	24.07	23.40	22.29	24.24	23.96	22.81	23.10
**Parameters**	**Exp. 15**	**Exp. 16**	**Exp. 17**	**Exp. 18**	**Total**	**Average**	**Std**
Mission success rate (%)	O	O	O	O	**88.9%**	-	-
Target’s travel distance (m)	26.68	28.17	28.08	26.37	444.89	24.72	9.01
Robot’s travel distance (m)	25.80	26.83	25.35	23.86	450.80	25.04	4.06
Robot’s travel time (s)	44.06	42.15	41.72	39.87	797.12	44.28	6.36
Robot’s average velocity (m/s)	0.59	0.64	0.61	0.60	-	0.57	0.11
# of frames	984	1029	940	948	18,563.0	1031.3	126.78
Successfully tracked (frames)	956	1003	923	908	16,857.0	936.5	141.83
Lost track of the target (frames)	28	26	17	40	1706.0	94.8	213.28
Successfully tracked (s)	42.81	41.09	40.96	38.18	722.27	40.13	5.86
Lost track of the target (s)	1.25	1.07	0.75	1.68	74.85	4.16	9.57
Lost track of the target	2.8	2.5	1.8	4.2	-	8.02	0.16
Successful tracking rate (%)	97.2	97.5	98.2	95.8	-	**91.98**	0.16
Computation time (fps)	22.33	24.41	22.53	23.78	-	23.34	0.85

std: standard deviation, fps: Frame per Second.

**Table 2:** Overall experimental results without other people.

Parameters	Exp. 1	Exp. 2	Exp. 3	Exp. 4	Exp. 5	Exp. 6	Exp. 7
Mission success rate (%)	O	O	O	O	O	O	x
Target’s travel distance (m)	32.931	27.401	28.906	28.005	30.94	28.819	0.00
Robot’s travel distance (m)	30.152	26.649	27.455	27.162	27.931	27.522	14.641
Robot’s travel time (s)	77.567	52.433	64.566	61.687	71.067	62.706	44.984
Robot’s average velocity (m/s)	0.39	0.51	0.43	0.44	0.39	0.44	0.33
# of frames	1699	1341	1537	1537	1913	1502	1153
Successfully tracked (frames)	1280	805	624	727	663	840	214
Lost track of the target (frames)	419	536	913	810	1250	662	939
Successfully tracked (s)	58.438	31.475	26.213	29.178	24.630	35.069	8.349
Lost track of the target (s)	19.129	20.958	38.353	32.509	46.437	27.637	36.635
Lost track of the target rate (%)	0.25	0.40	0.59	0.53	0.65	0.44	0.81
Successful tracking rate (%)	0.75	0.60	0.41	0.47	0.35	0.56	0.19
Computation time (fps)	21.90	25.58	23.81	24.92	26.92	23.95	25.63
**Parameters**	**Exp. 8**	**Exp. 9**	**Exp. 10**	**Exp. 11**	**Total**	**Average**	**Std**
Mission success rate (%)	O	O	O	O	**90.91%**	-	-
Target’s travel distance (m)	28.562	27.287	27.164	26.977	286.992	26.09	8.84
Robot’s travel distance (m)	27.435	26.492	26.345	26.214	287.998	26.18	3.98
Robot’s travel time (s)	71.974	59.975	51.331	55.327	673.617	61.24	9.84
Robot’s average velocity (m/s)	0.38	0.44	0.51	0.47	-	0.43	0.06
# of frames	1309	1333	1142	1096	15562	1414.73	251.87
Successfully tracked (frames)	1043	833	924	863	8816	801.45	266.45
Lost track of the target (frames)	266	500	218	233	6746	613.27	334.73
Successfully tracked (s)	57.348	37.479	41.532	43.565	393.276	35.75	14.51
Lost track of the target (s)	14.626	22.496	9.799	11.762	280.341	25.49	11.85
Lost track of the target rate (%)	0.20	0.38	0.19	0.21	-	0.42	0.21
Successful tracking rate (%)	0.80	0.62	0.81	0.79	-	**0.58**	0.21
Computation time (fps)	18.19	22.23	22.25	19.81	-	23.20	2.63

std: standard deviation, fps: Frame per Second.

## Abbreviations

The following abbreviations are used in this paper:

FRP	Freezing Robot Problem
RGB-D	Red, Green, Blue-Depth
HSV	Hue, Saturation, and Value
FoV	Field of View
SSD	Single Shot Detector
GPU	Graphics Processing Unit
CPU	Central Processing Unit
LOP	Last Observed Position
SLAM	Simultaneous Localization and Mapping
ROS	Robot Operating System
CNNs	Convolutional Neural Networks
YOLO	You Only Look Once
R-CNN	Regions with Convolutional Neural Network
SIFT	Scale Invariant Feature Transformation
HOG	Histograms of Oriented Gradient
ROI	Region of Interest
PID	Proportional Integral Derivative
WC	Walking Counter
Exp.	Experiment
Elev.	Elevator
lab	Laboratory
ADHD	Attention-Deficit Hyperactivity Disorder
fps	Frame per Second
